# Giant Parotid Pleomorphic Adenoma with Atypical Histological Presentation and Long-Term Recurrence-Free Follow-Up after Surgery: A Case Report and Review of the Literature

**DOI:** 10.1155/2020/8828775

**Published:** 2020-08-31

**Authors:** Mohammed AlKindi, Sundar Ramalingam, Lujain Abdulmajeed Hakeem, Manal A. AlSheddi

**Affiliations:** ^1^Department of Oral and Maxillofacial Surgery, College of Dentistry, King Saud University, Riyadh, Saudi Arabia; ^2^Department of Basic Sciences, College of Dentistry, Princess Nourah Bint Abdulrahman University, Riyadh, Saudi Arabia

## Abstract

Salivary gland tumors (SGT) comprise 3% of all head and neck tumors, are mostly benign, and arise frequently in the parotid gland. Pleomorphic adenoma (PA) is the commonest SGT, representing 60-70% of all benign parotid tumors. Clinically, parotid PA presents as irregular, lobulated, asymptomatic, slow-growing preauricular mass, involving both superficial and deep lobes, and could grow to gigantic proportions. Histologically, PA has epithelial and mesenchymal elements in chondromyxoid matrix and is managed surgically. Based on a review of 43 cases reported in English literature since 1995, giant parotid PA is reported as large as 35 cm (diameter) and 7.3 kg (resected weight). Although rare, 10 cases of malignant transformation were reported in the review. Surgical management included extracapsular dissection (ECD), superficial parotidectomy, and total parotidectomy for benign tumors, and adjuvant radiation or chemotherapy for malignant tumors. We further present the case of a 36-year-old healthy male with slow-growing and asymptomatic giant parotid PA, of 4-year duration. The patient presented with firm, lobulated preauricular swelling, provisionally diagnosed as PA based on radiographic and cytological findings. The tumor was resected through ECD, and the patient had uneventful postoperative recovery and a 7-year recurrence-free follow-up period. Histological examination revealed epimyoepithelial proliferation punctuated by chondromyxoid areas, with extensive squamous metaplasia and keratin cysts. To the best of knowledge from indexed literature, giant parotid PA is rarely reported in Saudi Arabia. In addition to its rarity, this case is reported for its benign nature despite atypical histological presentation, successful surgical management without complications, and long-term recurrence-free follow-up. Based on this report, clinicians must be aware of atypical histological presentations associated with PA and plan suitable surgical management and follow-up to avoid morbidity. Nevertheless, attempts must be made to diagnose and manage these lesions at an early stage and before they reach gigantic proportions.

## 1. Introduction

Neoplastic lesions of the salivary glands are uncommon and comprise less than 3% of all reported head and neck tumors [1, 2]. Nearly 80% of the reported salivary gland tumors (SGT) are benign and occur predominantly in major salivary glands, with the parotid gland being the commonest site (70–80%) [2, 3]. Often presenting as slow-growing, painless masses, tumors involving major salivary glands are rarely aggressive or malignant (<10%). On the contrary, tumors of minor salivary glands while occurring rarely have a preponderance to be malignant (80–90%) [2, 3]. Pleomorphic adenoma (PA) is the commonest SGT, accounting for almost 60–80% of all benign SGT and 60–70% of all parotid gland tumors [1, 3]. Clinically, PA presents as an irregular, rubbery, lobulated, slow-growing mass without any associated pain or discomfort. The presenting complaint is typically related to unpleasant or unesthetic facial appearance, which when disregarded can lead to patients reporting with huge lesions [4]. Reports in the literature suggest resected dimensions of PA to be frequently in the range of 2 cm to 6 cm and rarely reaching even up to 25–35 cm [4, 5].

As the name suggests, PA is histologically categorized as a benign mixed (pleomorphic) tumor of ductal and myoepithelial cell origin. Owing to the pluripotential nature of myoepithelial cells, the tumor is composed of epithelial and fibrous, myxoid, and cartilaginous mesenchymal elements surrounded by a pseudocapsule, with occasional squamous metaplasia [3, 4, 6]. While the diagnosis of PA is based primarily on clinical and histological findings, the mainstay of management is by surgical excision [1, 4]. Depending upon their size and depth of involvement, parotid PA is surgically managed either by superficial parotidectomy (SP), extracapsular dissection (ECD), or total parotidectomy (TP). All of the above procedures carry the risk of postoperative facial nerve paralysis and Frey's syndrome [1, 7]. Recurrence is usually associated with inadequate clearance and incomplete removal of pseudocapsule, and malignant transformation has been reported with large, long-standing PA [4, 7].

Although uncommon, giant pleomorphic adenomas of the parotid gland have been reported. An electronic search of the English-language articles through the Medline, Scopus, and Google Scholar databases revealed 43 reports of giant parotid PA since 1995 [4, 8–32], with sizes ranging up to 28-33 cm [15, 18, 27] and tumor mass ranging up to 6.85–7.3 kg [10, 27]. Major reasons for patients reporting with large PA are lack of resources, inaccessibility to medical facilities, fear of surgical procedure, and poor awareness, compounded by an asymptomatic and slow-growing lesion [4]. The aim of this paper is to add to the existing scientific literature, a case of giant parotid PA treated surgically by ECD, followed by uneventful postoperative recovery and a 7-year recurrence-free follow-up. This paper also attempts to highlight the benign nature of parotid PA, despite the atypical histological presentation, which could be associated with it.

## 2. Case Report

A 36-year-old healthy male patient reported to the oral and maxillofacial surgery outpatient clinic at the College of Dentistry and Dental University Hospital, King Saud University, in October 2012. The patient sought medical attention for a slow-growing, painless swelling in the right preauricular region. History revealed that the patient noticed the swelling almost 4 years ago, and since then, it had gradually increased in size with no obvious symptoms or changes to the overlying skin. Upon interviewing, the patient reported no relevant medical or surgical history and mentioned fear of surgery and absence of discomfort as reasons for delaying medical consultation, in spite of an unesthetic facial appearance.

**Figure 1:**
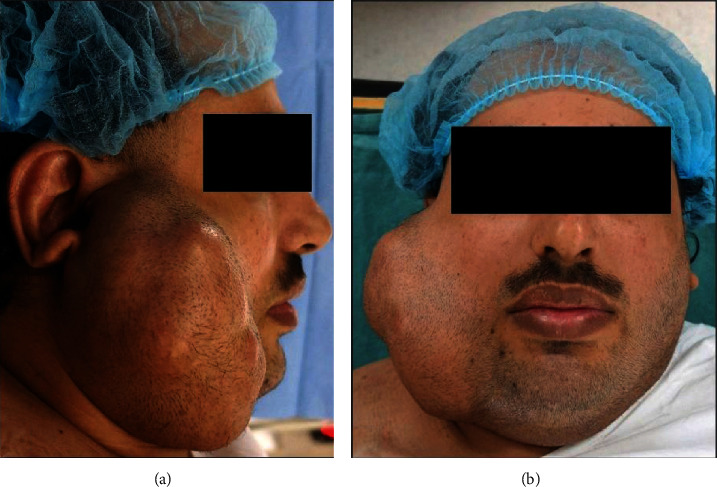
Preoperative clinical photograph of the right preauricular swelling. (a) Right lateral facial view shows the swelling extending superoinferiorly from a point anterior to the helix of the external ear until the lower border of the mandible; anteroposteriorly, the swelling is seen extending from the angle of the mouth to the posterior border of the mandible; the ear lobe is deflected outward and elevated, and the skin overlying the swelling appears free of any ulceration, puckering, or discharge. (b) Frontal facial view shows the swelling causing facial asymmetry and obliterating the view of most of the right external ear; there is no clinical evidence of facial nerve weakness or deficit.

**Figure 2:**
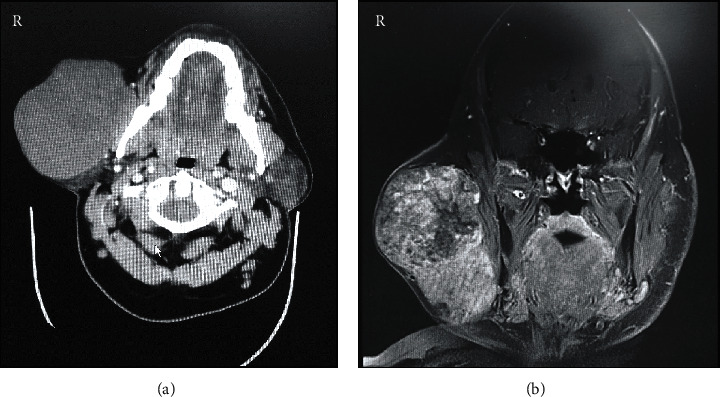
Preoperative radiographic examination of the right preauricular swelling. (a) Contrast-enhanced computed tomography axial section at the level of mandibular teeth shows a well-defined mass lesion in the superficial lobe of the right parotid gland, without any underlying bony erosion and normal-appearing pharynx, larynx, and parapharyngeal spaces. (b) Magnetic resonance imaging coronal section along the posterior border of the mandible shows a large, heterogeneous, well-demarcated solid mass lesion within the right parotid superficial lobe and measuring 10 × 7 × 8 cm at maximum dimensions.

Clinical examination revealed a firm, nontender, nodular, and mobile swelling with apparently normal overlying skin. The swelling extended superoinferiorly from the level of the external ear to the lower border of the mandible and anteroposteriorly from the angle of the mouth to the posterior border of the mandible. There was no lymph node involvement or facial nerve deficit (Figure 1). Preoperative computed tomography (CT), magnetic resonance imaging (MRI), and fine-needle aspiration cytology (FNAC) were ordered. CT with contrast revealed a well-defined mass lesion in the superficial lobe of the right parotid gland, without any underlying bony erosion and normal-appearing pharynx, larynx, and parapharyngeal spaces. While confirming the CT findings, head and neck MRI further demonstrated a well-demarcated, heterogeneous, mass lesion measuring 10 × 7 × 8 cm in maximum dimension (Figure 2). FNAC showed numerous scattered groups and clusters of plasmacytoid myoepithelial cells, associated with a chondromyxoid matrix. A provisional diagnosis of PA with no malignant tendency was arrived at based on CT, MRI, and FNAC findings.

**Figure 3:**
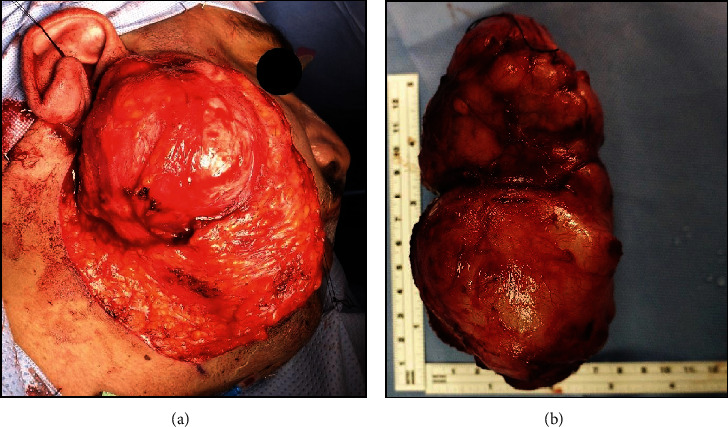
Intraoperative photograph showing (a) the surgical plane for extracapsular dissection of the right parotid tumor and (b) the excised tumor specimen.

Surgical removal of the right parotid SGT under general anesthesia was planned and explained to the patient. Following informed consent, the lesion was excised completely through ECD, with preservation of all branches of the facial nerve. The right parotid gland was approached using a cervically extended preauricular skin incision. A clearly discernible plane of dissection around the tumor was used for dissecting the tumor mass, without any iatrogenic damage to the facial nerve branches. Owing to the long-standing nature, multiple small feeder vessels had to be ligated circumferentially around the tumor to achieve hemostasis. The intraoperative period was unremarkable, and the patient did not require any blood transfusions (Figure 3). The resected mass was bilobed and ovoid in shape with a final dimension of 7 × 13 × 7 cm and weighing 1.2 kg.

**Figure 4:**
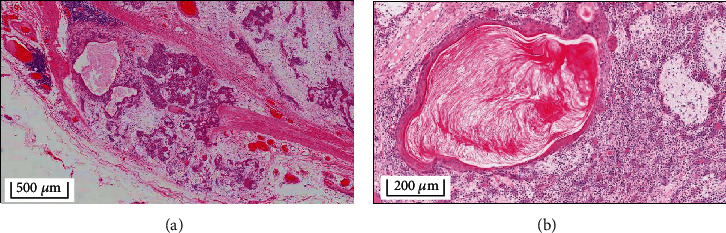
Histological examination of the excised tumor specimen showing (a) a partially encapsulated mass lesion containing myoepithelial and ductal proliferation, with stromal hyalinization, squamous metaplasia, and keratinization; epithelial islands exhibiting papillary configuration, large cysts surrounded by inflammation, focal areas of chondromyxoid changes and fibrosis, and tumor islands approaching and penetrating the capsule are evident (HE original magnification ×4); (b) extensive squamous metaplasia and keratin cyst formation are conspicuous at higher magnification (HE original magnification ×10).

Histopathological examination of the excised specimen gave a gross appearance of a partially encapsulated mass containing myoepithelial and ductal proliferation. There was marked stromal hyalinization, squamous metaplasia, and keratinization. Some epithelial islands exhibited papillary configuration, along with large cysts and inflammation. Chondromyxoid changes and fibrosis were evident throughout the tumor. Certain foci of tumor islands were seen approaching and breaking through it. Hematoxylin and eosin (H&E) stained sections revealed a partially encapsulated tumor with variable histopathological features and focal effacement of the fibrous capsule. The tumor typically showed epithelial/myoepithelial proliferation punctuated by chondromyxoid areas. Aggregates of plasmacytoid myoepithelial cells as well as ducal differentiation surrounded by clear myoepithelial cells were evident. Based on the above findings, a final diagnosis of benign PA was reached. While the tumor sections showed no evidence of malignant change, there was extensive squamous metaplasia and keratin cyst formation, which were atypical for PA (Figure 4). The patient was therefore advised close follow-up, once every month for the first year postoperatively and subsequently once in six months.

**Figure 5:**
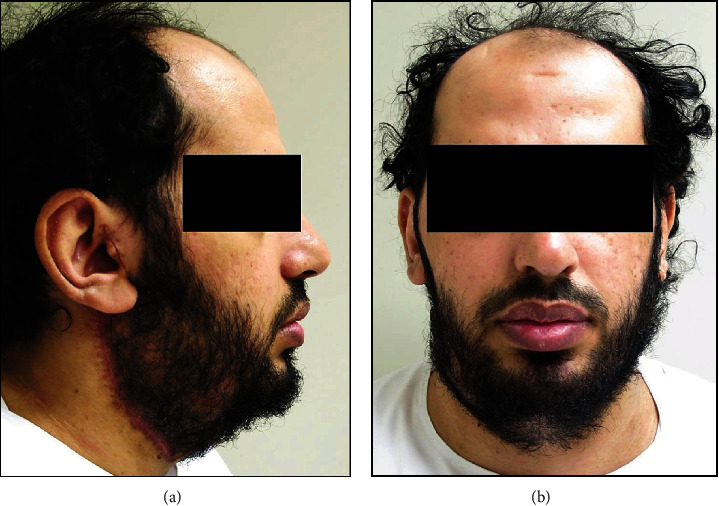
Postoperative clinical photograph taken 6 weeks postsurgery. (a) Lateral facial view shows healing surgical incision without any obvious postoperative sequelae. (b) Symmetric facial appearance observed in the frontal facial view, with no clinical weakness of muscles of facial expression.

**Figure 6:**
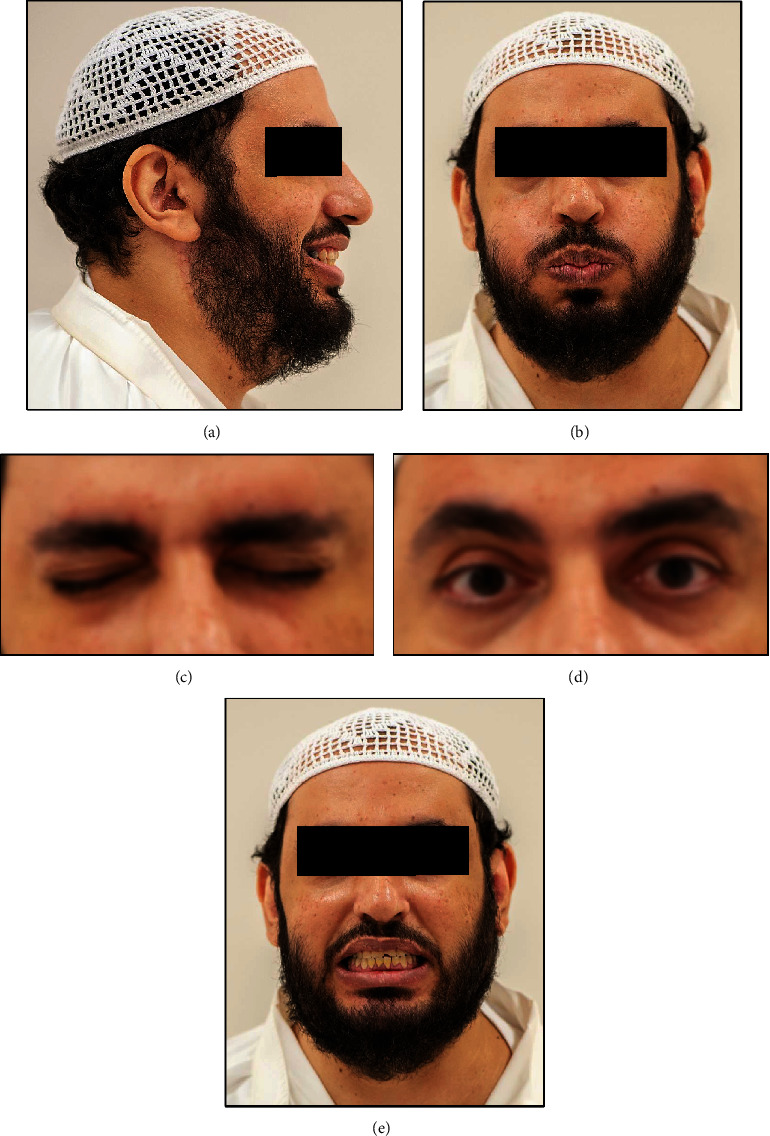
Postoperative clinical photograph taken 7 years postsurgery with facial gestures eliciting unrestrained action of different muscles of facial expression. (a) Unremarkable healing of the surgical wound without any scarring and the patient is seen smiling. (b) Frontal facial view showing symmetric appearance and the patient is seen puffing the cheeks. (c, d) Bilateral symmetric eyelid closure and opening. (e) The patient is seen grinning broadly.

At 6 weeks postsurgery, the patient had unremarkable wound healing without any neurological deficit of the facial nerve (Figure 5). As of December 2019, the patient had a recurrence-free follow-up period of 7 years and presented with normal activity of muscles of facial expression, indicating the absence of any long-term facial nerve weakness (Figure 6).

## 3. Discussion

The parotid gland is the largest salivary gland with an average weight ranging from 0.015 to 0.021 kg and measuring approximately 5.8 × 3.4 cm in the craniocaudal and ventrodorsal dimensions, respectively. Being the first salivary gland to develop in utero, during the 6^th^ gestational week, it is anatomically located bilaterally between the mastoid process of temporal bone and ramus of the mandible. The terminal branches of the facial nerve are an important anatomic landmark which divide the parotid gland into its superficial and deep lobes [33]. Although SGT are uncommon, they are predominantly benign and are reported frequently in the parotid gland [2]. The present report details a case of giant PA in the right parotid gland, along with its surgical management, histological presentation, and long-term recurrence-free follow-up.

Pleomorphic adenoma is the commonest mixed SGT arising in the parotid gland, and several cases have been reported in the literature. Although there are no specific physical criteria outlined in the literature to classify giant parotid PA, the earliest recorded case report dates back to 1863 [27]. In this report, Spence reported a mixed tumor involving lateral face and neck and a resected mass weighing greater than 1 kg [27]. Similarly, Short and Pullar (1956) reviewed and reported a case of giant parotid PA weighing about 2.3 kg [27]. In a report reviewing 31 cases of giant parotid PA over a period of 140 years by Schultz-Coulon, the resected tumor weights ranged from 1.0 to 26.50 kg, with a greater female predilection (64.5%) and only 3 cases of malignant transformation [15]. Based on a review of the 10 largest parotid PA published between 1863 and 1994, Buenting et al. reported resected tumors ranging in weight from 2.83 to 26.50 kg, in patients with age ranging from 25 to 85 years and 90% female predilection [10].

A literature search was conducted to review giant parotid PA cases reported in Medline, Scopus, and Google Scholar databases. The search strategy involved a combination of search keywords including “GIANT”, “PAROTID GLAND”, and “PLEOMORPHIC ADENOMA”, based on which 288 articles were identified from the three databases (Medline—66; Scopus—63; Google Scholar—159). Reviewing their abstracts, articles published in English only were selected based on them reporting a case or series of cases of giant parotid PA, including clinical, radiographic, histological, and surgical outcomes. Twenty-six articles published in English language were identified [4, 8–32] since 1995, and they reported 43 cases of PA in total. While most of the articles selected for review were single case reports, three articles were case series reporting about two cases [16], three cases [17], and 15 cases [32], respectively. The largest case series in the present review, comprising 15 PA patients, was reported by Pareek et al. [32].

Although majority of the reported cases were in patients aged 45 years or older (*n* = 30, 69.8%), the age at clinical presentation and surgery ranged from 21 to 92 years. The asymptomatic, slow-growing nature of pleomorphic adenoma was evidenced by the fact that the duration from the first observation of lesion to reporting for treatment varied from 5 to 35 years. Demographically, there were more females (*n* = 25, 58.1%) than males, and all reported cases were unilateral, with the right side (*n* = 23, 53.5%) affected more than the left. In terms of clinical dimension, the tumors ranged from 3 to 5 cm in diameter [16, 17], until 35 × 28 cm [27], in two perpendicular planes. The clinical dimensions corroborated with the resected tumor weight, wherein the smallest tumor weighed 0.12 kg [16] and the largest weighed 7.3 kg [27]. Predominant clinical presentation of the tumors was that of a large, lobulated, and pedunculated mass with apparently normal overlying skin. Ulceration of the skin was reported in eight patients, out of which five were reportedly associated with malignant change [15, 23, 24, 30, 32], and the remaining three were due to injury [18, 29, 32]. Anatomically, the tumor more commonly involved the superficial lobe of the parotid gland (*n* = 29, 70.7%) giving rise to the clinical presentation of a large preauricular mass. Nevertheless, when the deep lobe of parotid and parapharyngeal spaces were involved by the tumor, patients presented with an intraoral swelling leading to soft palate displacement, difficulty in swallowing and breathing, and obstructive sleep apnea [11, 13, 16, 17, 22, 25]. While diagnosis was primarily based on clinical and radiographic (USG, CT, and MRI) findings, preoperative diagnosis was established through FNAC in most cases. The clinical, radiographic, surgical, and histological findings in the reviewed case reports are detailed in Table 1.

Preoperative diagnosis of PA is routinely based on clinical findings, supplemented by radiological investigations such as CT, MRI, and USG [2]. The role of FNAC in arriving at a provisional diagnosis has been debated and considered nonrepresentative due to varying histological patterns at different sites within the same tumor [34]. In terms of histopathological diagnosis, the characteristic feature of PA is its histological diversity and differing arrangements of epithelial and mesenchymal tissue elements. Das and Anim [34], based on a study comparing FNAC and histological sections in PA, reported consistent findings of epithelial cells in a myxoid stroma through cytological and histological examination. Nevertheless, they reported better characterization of oncocytic changes such as acini, giant cell and mucus globule formation, and squamous and chondroid metaplasia in histological sections [34]. Preoperative diagnosis of PA in the present case was based on a combination of clinical examination, radiographic investigations, and FNAC. Our radiographic finding (CT and MRI) of well-demarcated, lobulated, and heterogeneous mass lesion involving the parotid gland and FNAC finding of plasmacytoid epithelial cells in a chondromyxoid matrix were in coherence with the majority of cases reported in the review (Table 1). Additionally, necrotic changes [10, 24, 30] and calcifications [4, 30], within the tumor, have also been reported in the literature, based on CT and in association with malignant change [24, 30]. The combination of CT and MRI enables optimum preoperative diagnosis of pleomorphic adenomas and precise planning of the surgical approach for tumor resection [11]. In addition to volumetric information, CT with contrast provides knowledge about vascularity of the tumor and MRI shows the relationship of the tumor to surrounding vital structures in the head and neck regions [11].

The treatment of PA irrespective of their size, severity, or malignant potential is only by surgery. Based on our literature review, giant PA involving the superficial lobe of parotid was managed either by SP or ECD [4, 10, 24, 26–29, 31, 32]. On the contrary, TP was reportedly done for tumors exhibiting malignant characteristics [8, 12, 15, 17, 19, 20, 23, 30] and those involving both the deep and superficial lobes [9, 13, 14, 16–18, 21, 32] (Table 1). Since the present case was that of a PA involving superficial parotid only (Figures 1 and 2), the patient was surgically managed through ECD (Figure 3), avoiding any iatrogenic damage to the facial nerve (Figure 5). The surgical techniques for resecting PA have evolved over the last century and have essentially focused on preserving anatomic and functional integrity of the facial nerve and its branches [1, 7]. While intracapsular removal was advocated, during the late 19^th^ and early 20^th^ centuries in an attempt to avoid facial nerve injury, the technique was abandoned due to its high risk of recurrence (≥45%) [1, 7]. This leads to the popularity of SP, associated with a very low recurrence rate (≤2%), but still carrying a risk of facial nerve injury, Frey syndrome, and loss of facial contour [1]. In the last 2-3 decades, ECD has gained significance as a surgical technique which involves tumor resection along with the capsule and a thin rim of extracapsular normal tissue, without attempting to identify or dissect the facial nerve and its branches [1]. Based on literature reviews, Kato et al. [1] and Bonavolontà et al. [7] reported that ECD is safe, is economical, and reduces operating time and risk of morbidity. They further considered it as the treatment of choice for mobile, benign tumors involving the superficial lobe of the parotid gland [1, 7]. Moreover, traction due to gravity in giant PA causes the tumor mass to descend away from the facial nerve and its branches, thereby facilitating ease of dissection, as encountered in the present case [10].

Several surgical approaches to remove PA and other benign parotid gland tumors involving the deep lobe and extending to the lateral pharyngeal and parapharyngeal spaces have been reported [11]. These include cervical, cervical-transpharyngeal, infratemporal fossa, parotid-cervical, transoral, and transparotid approaches. In the present review, 9 cases of PA were reportedly involving the deep lobe of parotid and extending into pharyngeal spaces. The cervical-transparotid surgical approach was used for resection in 6 cases [11, 13, 16, 17, 22], a transcervical, split-mandibulotomy approach was used in 2 cases [17, 25], and one case was managed through a transoral approach using “Double-Y” incision in soft palate [16] (Table 1). Postoperative complications following surgical resection of parotid PA are similar to that of other benign SGT [1, 4, 7, 23]. These include hemorrhage, hematoma, seroma, fistula, hypoesthesia, scarring, Frey syndrome, transient facial nerve injury, and facial paralysis [7]. While these complications are reportedly associated with all surgical techniques mentioned before, ECD was associated with a significantly lower risk of hypoesthesia, Frey syndrome, and facial nerve injury and paralysis [7]. Nevertheless, when dealing with large parotid tumors with suspected malignancy, which mandate identification and dissection of facial nerve branches, no significant differences were observed in the outcomes and complications between antegrade and retrograde dissection techniques [30]. Postoperative complications reported in the current review include reactionary hemorrhage immediately after surgery [4] and transient facial nerve deficit which recovered in durations ranging from one month to 6 months [4, 11, 14, 16, 17, 32] (Table 1). Although there were no documented cases of permanent facial nerve injury, Calvo-Henriquez et al. [28] reported a case of preoperative “House-Brackman Grade III” facial nerve deficit, which persisted postoperatively.

In addition to the long-standing nature of the tumor and its large size, the currently reported case also presented with atypical histological findings. While the histological picture was predominantly coherent with that ofPA characterized by epithelial tissue dispersed in a chondroid and myxoid matrix [34], there was marked squamous metaplasia and keratin cyst formation (Figure 4). These findings are unusual in PA of the parotid gland and lead to a high index of suspicion towards a diagnosis of mucoepidermoid carcinoma or squamous cell carcinoma [35]. However, similar findings have been reported in the literature in PA arising in labial and palatal minor salivary glands, wherein malignant transformation is highly suspected and ranges from 2 to 23% [6, 35]. Squamous metaplasia of glandular cells is attributed to hypoxia within the growing tumor and combined with keratin cysts; they mimic cutaneous appendages and are termed as “cystic pleomorphic adenoma with extensive adnexa-like differentiation” [35]. It has further been emphasized that the above findings during the histological examination of suspected pleomorphic adenomas warrant meticulous histopathological evaluation to avoid surgical overtreatment [6, 35]. Analyzing the histological findings in the present review, only one case was associated with cartilaginous metaplasia [10], without any evidence of malignant change, similar to that observed in our case too. Nevertheless, malignant transformation was reported based on final histopathological examination in 10 cases, including 5 cases of carcinoma expleomorphic adenoma (CA-ExPA) [17, 20, 23, 24, 32], one case of adenocarcinoma along with PA [15], 3 cases of mixed malignant tumor arising in PA [8, 12, 30], and one case of metastasizing PA with renal metastasis [19] (Table 1). Interestingly, one of the cases of CA-ExPA also had a malignant melanoma component, arising possibly from melanocytes within parotid tissue [20].

Malignant transformation of PA is reported at around 12% and comprises nearly 4% of all SGT [20]. According to the World Health Organization (WHO) classification of SGT, malignant tumors arising in PA were originally classified either into CA-ExPA or the relatively rare mixed malignant tumor with epithelial and mesenchymal components [8, 12]. While metastasizing PA, with metastatic lesions exhibiting histological characteristics of PA and no malignant change was originally considered a malignant variant, it has been relegated to the benign category as per the WHO classification (2017) of SGT [36]. Although rare, metastasizing PA is frequently associated with multiple recurrences of the primary tumor. Bhutta et al. [19] reported a case of metastatic renal PA, with multiple postsurgical recurrences of the parotid PA in over a decade. Based on a retrospective study of 221 patients with PA and 15 patients with CA-ExPA, Seok et al. [37] reported increasing age, regional lymph node involvement (>5 mm), and MRI apparent diffusion coefficient as the distinguishing clinical and radiographic features of CA-ExPA. In terms of histology, cellular atypia and stromal hyalinization have been regarded as the key delineating features of CA-ExPA from benign PA [36, 38]. Based on immunohistochemistry (IHC), loss of expression of PLAG-1 (pleomorphic adenoma gene-1) protein and overexpression of Ki-67, p53, and HER-2 (human epidermal growth factor receptor-2) are considered suggestive of malignant transformation in otherwise benign PA [38, 39]. At a molecular genetic level, malignant transformation of PA is further characterized by PLAG-1/HMGA-2 (High Mobility Group AT-Hook 2) fusion and rearrangement, p53 mutation, and HER-2 amplification [36, 38, 39].

In the present review, PA with malignant transformation was treated surgically, followed by radiotherapy for locoregional disease and chemotherapy for distant metastases. Within postoperative follow-up periods ranging from 6 months to 5 years, only one case of recurrent benign PA was reported in the review [19]. Among tumors with malignant change, locoregional recurrence was reported in one case of mixed malignant tumor [8], cervical lymph node metastasis was seen in 2 cases [12, 20], and distant metastasis to the anterior chest wall, lungs, liver, pelvis, and kidney was observed in 5 cases [12, 15, 19, 20, 30]. While there was no mortality associated with benign PA or its surgical treatment, 3 patients with malignant transformation died within 6 months after surgery as a result of metastatic disease [8, 15, 20]. The longest recurrence-free follow-up for a giant parotid PA in the present review was 5 years [27], and this was still lesser than the 7-year recurrence-free follow-up observed in our case.

## 4. Conclusion

Giant parotid PA is rare, and to the best of our knowledge from indexed literature, this is one of the very few cases reported in this geographical region (Saudi Arabia). Apart from its rarity, the present case is being reported for its long-standing clinical presentation, successful surgical management through ECD without any postoperative complications or facial nerve deficit, atypical histological presentation, and long-term recurrence-free follow-up. In addition to complete tumor removal, ECD helped preserve the integrity of facial nerve branches. Interestingly, the present case of giant parotid PA was benign in spite of associated squamous metaplasia and keratin cyst formation identified histologically in the resected specimen. Nevertheless, this mandated a long-term follow-up of the patient. Therefore, based on this case report, clinicians must be aware of atypical histological presentation associated with parotid PA and plan suitable surgical management and follow-up, to achieve complete tumor removal and avoid morbidity. Lastly, attempts must be made to diagnose and manage PA at an early stage and before they reach gigantic proportions, through better public health care initiatives and creating awareness among all physicians and patients.

## Figures and Tables

**Table 1:** Review of giant parotid pleomorphic adenoma case reports and their demographic, clinical, radiographic, surgical, and histological findings.

Author (year)	Patient demographics			Preoperative evaluation			Surgical intervention			Postoperative period		
	Age (in years)/gender	Duration of lesion	Affected side	Clinical dimension	Clinical presentation	Investigations	Procedure	Resected dimension	Reason for surgery	Histological findings	Postoperative course	Follow-up
Alvarez-Cañas and Rodilla (1996) [8]	86/F	15 years	Left		Large painless preauricular mass which enlarged suddenly over the past 1 year and associated with facial nerve deficit	Only clinical examination	Total parotidectomy	9.5 × 8 × 7 cm	Sudden increase in size with facial nerve deficit	Mixed malignant transformation of PA with salivary ductal carcinoma and high-grade fibrosarcoma elements		Patient developed local recurrence of tumor and died 6 months after surgery

Lomeo (1996) [9]	74/F	35 years	Left		Large preauricular mass	Only clinical examination	Total parotidectomy		Patient was convinced for surgery by grandchildren	Pleomorphic adenoma		

Buenting et al. (1998) [10]	85/F	20 years	Right		Large, multinodular preauricular mass with evidence of infection. The mass was tensely cystic and had prominent veins near the base	CT showed a parotid mass 14 cm across with extensive necrotic foci and numerous feeding vessels, which were not amenable to embolization	Extracapsular dissection of the tumor mass	26 cm diameter (6.85 kg)	Inadvertent injury to the base of the mass resulting in bleeding and infection	Pleomorphic adenoma with extensive necrosis and cartilaginous metaplasia		1-year recurrence-free follow-up

Rodriguez-Ciurana et al. (2000) [11]	48/F	30 years	Right		Large mass in the submandibular and laterocervical regions, extending intraorally from soft-palate to floor of mouth	MRI showed a mass involving the deep lobe of the parotid gland, extending into parapharyngeal, prestyloid, and submandibular spaces, displacing external and internal carotid arteries and thinning the ramus of mandible. Measuring 6 × 5 × 4 cm. FNAC was indicative of PA	Deep lobe parotidectomy through cervical transparotid approach			Pleomorphic adenoma	Transient facial nerve weakness for 4 weeks	

Manuel (2002) [12]	68/F		Left		Recurrent parotid mass which was incompletely excised earlier and diagnosed as mixed malignant tumor		Total parotidectomy, with removal of facial nerve branches due to tumor infiltration and modified neck dissection		Recurrent lesion in the previously excised tumor site	Carcinosarcoma arising from PA, with residual PA, epimyoepithelial carcinoma, and pleomorphic sarcoma. Multiple metastatic cervical lymph nodes		Patient was operated for metastatic anterior chest wall mass, 7 months postsurgery and had an 18-month disease-free follow-up
Panoussopoulos et al. (2002) [13]	63/M	30 years	Left	13 × 12 cm	Large, lobulated mass in the submandibular, preauricular, and laterocervical regions, extending intraorally to the lateral pharyngeal wall at the level of tongue	MRI showed a well-defined mass involving both superficial and deep lobes of the parotid gland, and extending into the parapharyngeal space, displacing tissues deep to the tonsil	Total parotidectomy through cervical transparotid approach			Pleomorphic adenoma		

de Silva et al. (2004) [14]	76/M	>30 years	Left	20 × 30 cm	Large, oval preauricular swelling, firm in consistency, with venous engorgement on overlying skin and movable	FNAC was indicative of PA	Total parotidectomy with preservation of facial nerve	20 × 14 × 12 cm (3.5 kg)		Pleomorphic adenoma	Facial nerve deficit observed 1 week postoperatively and recovered 90% by 1 month	1-year recurrence-free follow-up and complete recovery of facial nerve function

Honda et al. (2005) [15]	72/F	20 years	Left		Large, pedunculated preauricular mass extending up to the submandibular region, with a history of rapid growth in preceding 3 months and 2 areas of ulceration with yellowish, foul-smelling discharge in the lower part of the mass. An associated anterior chest wall mass measuring 10 × 8 cm was clinically identified	CT showed a mass with multiple encapsulated nodules involving the entire parotid gland, having several feeder vessels and supplied predominantly by the transverse facial artery. Coincidental finding of abnormal skull base lesion measuring 4 cm in diameter. Chest radiograph revealed multiple metastatic nodules, measuring around 1 cm, in both lungs	Total parotidectomy + simultaneous resection of anterior chest wall mass	33 × 18 × 17.5 cm exophytic tumor (6.051 kg)	Sudden increase in size with ulceration and discharge	Pleomorphic adenoma with focal areas of malignant adenocarcinoma cells with hyperchromatic nuclei and increased mitotic figures. Similar histological findings observed in the resected anterior chest wall mass		Patient died 6 months postsurgery, due to metastatic lung disease

Ruiz-Laza et al. (2006) [16]	54/M	5 years	Left	3 cm	Solid mass in preauricular and mandibular angle regions	MRI showed a multilobulated mass measuring 8 cm in diameter and extending from deep lobe of the parotid gland into parapharyngeal space, displacing the pharyngeal airway medially and the jugular and carotid vessels posteriorly. FNAC was indicative of PA	Total parotidectomy through cervical transparotid approach and facial nerve preservation			Pleomorphic adenoma	Postoperative facial nerve deficit which recovered completely in 6 months	3-year recurrence-free follow-up

Ruiz-Laza et al. (2006) [16]	21/M		Right		Intraoral mass occupying the entire soft palate with no other associated symptoms	MRI showed a well-defined mass lesion measuring 6 × 5 × 4 cm in the parapharyngeal space and with apparent continuity to the deep lobe of the parotid gland. FNAC was indicative of PA	Surgical excision of tumor mass only, through intraoral approach and “Double-Y” incision in soft palate	11 × 10 cm (0.12 kg)		Pleomorphic adenoma		3-year recurrence-free follow-up

Sergi et al. (2008) [17]	36/M	1 year	Left	5 cm	Solid preauricular mass	USG showed two hypoechogenic, lobulated masses measuring 2.5 × 1.9 × 1.6 cm in the deep lobe of the parotid gland and 4.4 × 5.1 × 4.6 cm posterior to mandibular ramus. MRI revealed expansive mass measuring about 5 cm in the deep lobe of the parotid gland, extending from mandibular angle to lateral pharyngeal wall medially	Using cervical transparotid approach, superficial and deep lobe parotidectomy performed separately to preserve facial nerve branches			Pleomorphic adenoma	Transient neurological deficit of marginal mandibular branch of facial nerve	

Sergi et al. (2008) [17]	42/M		Right	3 cm	Solid preauricular mass	MRI showed a mass in the deep lobe of the parotid gland, extending into parapharyngeal space and displacing the pharyngeal muscles medially. FNAC was indicative of PA	Using cervical transparotid approach, superficial and deep lobe parotidectomy performed separately to preserve facial nerve branches		Increased in size over 2 months	Pleomorphic adenoma		

Sergi et al. (2008) [17]	38/F		Left		No extraoral swelling. Intraoral mass lateral to the soft palate and displacing it across the midline	MRI showed inhomogeneous, expansive mass arising from the deep lobe of the parotid gland and measuring 5.5 × 5.5 cm in the lateral pharyngeal space. The mass was seen displacing the pterygoid and pharyngeal muscles medially. FNAC was indicative of PA	Separate superficial and deep lobe parotidectomy through transcervical, mandibular split approach to preserve facial nerve branches		Pain while swallowing and sensation of foreign body in the throat since 5 months	Pleomorphic adenoma with a nucleus of carcinoma ex-PA	Postoperative radiotherapy	

Takahama et al. (2008) [18]	78/M	>30 years	Right	30 cm	Large, multinodular preauricular mass extending to the submandibular region and crossing the midline. Focal areas of ulceration in the lower part of the mass	Only clinical examination	Total parotidectomy	28 × 20 × 16 cm (4.0 kg)		Pleomorphic adenoma		

Bhutta (2009) [19]	63/F		Left		Slow-growing mass in the left superficial parotid	Only clinical examination	Excision done in 1993, followed by multiple recurrences managed surgically through excision from 1995-2006			Early lesion was suggestive of PA. Recurrent lesions resembled PA with high mitotic rate and no malignancy	45 Gy external beam radiation therapy (in 25 fractions) given in 2000 to prevent recurrence	In 2006, CT showed a right kidney mass, diagnosed as metastasizing PA by histology (typical features of PA with positive Ki67 staining)

Karpowicz et al. (2010) [20]	45/M		Right		Subcutaneous parotid mass with ipsilateral cervical lymphadenopathy involving multiple nodes. Associated with severe pain and rapid increase in size. Clinically staged as stage Iva malignant disease	Only clinical examination	Total parotidectomy with comprehensive neck dissection	Nonencapsulated tumor measuring about 3.5 cm	Severe pain and rapid increase in size	Malignant epithelial cells in a chondromyxoid stroma indicative of carcinoma ex-PA. Malignant foci included high-grade squamous cell carcinoma and adenocarcinoma. One of the cervical lymph nodes showed evidence of metastatic carcinoma. Immunohistochemistry identified a melanoma component	Two months postsurgery, the patient reported severe pelvic pain, diagnosed as metastatic bone disease through MRI	Patient died 3 months postsurgery due to metastatic disease

Cetin et al. (2012) [21]	55/F	20 years	Left	15 × 15 × 20 cm	Large preauricular mass extending to cervical regions, with overlying skin atrophic and vascular	USG showed a lobulated mass in the parotid gland with both homogeneous and heterogeneous echotextures	Total parotidectomy			Pleomorphic adenoma		

Morariu et al. (2012) [22]	42/M		Right		Large mass arising from the lateral pharyngeal wall, displacing the soft palate and uvula, and narrowing the pharyngeal airway. Associated symptoms of painful swallowing, heavy snoring, and sleep apnea for past 1 year	MRI showed a circumscribed mass lesion 7 × 6 × 4 cm extending from the deep lobe of the parotid gland into the parapharyngeal space with fluid spaces and septation. CT angiogram while showing splayed internal and external carotid arteries, ruled out any abnormal vascularity. Transoral FNAC was indicative of PA	Deep lobe parotidectomy through transparotid approach		Pharyngodynia and nocturnal hypoxia symptoms	Pleomorphic adenoma	Patient reported relief from nocturnal hypoxia, snoring, and sleep apnea symptoms, postoperatively	6-month recurrence-free follow-up

Yoshida et al. (2013) [23]	40/F	17 years	Left		Small parotid swelling before 17 years, diagnosed as PA by FNAC. Surgery delayed for 10 years due to patient's fear and then lost to follow-up. Swelling grew rapidly in past 6 months, causing gait disturbance and skin ulceration with foul-smelling, bloody discharge from the lower part of lesion	CT showed a nodular mass arising from the parotid gland and attached in its deeper aspect to the carotid sheath. Evidence of metastasis in chest radiograph owing to bilateral hilar lymphadenopathy and coin-shaped radiolucency in the right lung. Incision biopsy was indicative of PA, with clinical suspicion of malignancy	Total parotidectomy and en bloc resection of the tumor along with the lower portion of the auricle	25 × 28 × 18 cm (4.80 kg)	Rapid growth of tumor in last 6 months with cervical and thoracic scoliosis and gait disturbance	Nearly 80% of the resected tumor sections showed evidence of PA. Sections of the tumor near the ulcerated areas showed undifferentiated malignant cells indicative of carcinoma ex-PA	Postoperative adjuvant chemotherapy for metastasis	6-month follow-up with no local recurrence

Pamuk et al. (2014) [24]	82/F	20 years	Right	13 × 13 × 10 cm	Large, multilobular preauricular mass with areas of ulceration and necrosis on overlying skin	CT showed a giant, exophytic mass in the superficial lobe of the parotid gland with multiple necrotic spaces and enhanced vascularity. Incisional biopsy was indicative of a salivary gland neoplasm without ruling out malignant transformation	Superficial parotidectomy with excision of overlying ulcerated skin	14 × 12 × 9 cm		Pleomorphic adenoma with multiple foci of neoplastic proliferation, along with cellular atypia and necrosis. Final diagnosis carcinoma ex-PA	Postoperative radiotherapy 60 Gy	Patient died 8 months postsurgery due to cerebrovascular accident

Datarkar and Deshpande (2014) [25]	40/F		Right		Large, firm intraoral mass arising from the lateral pharyngeal wall, displacing the soft palate and crossing midline. Associated symptoms of difficulty in swallowing and breathing, and sleep apnea for past 6 months	CT showed parapharyngeal space mass extending medially across the midline and laterally between the posterior border of ramus and styloid process. MRI showed lobulated, homogeneous mass lesion extending from the deep lobe of the parotid gland with hypointense septae and measuring 5.4 × 6.5 × 3.5 cm, and indenting on lateral pharyngeal wall. No involvement of skull base or intracranial extension was observed. Transoral FNAC was indicative of PA	Deep lobe parotidectomy through transcervical, mandibular split osteotomy approach	5.5 × 6.5 × 3.5 cm	Difficulty in swallowing and breathing, and sleep apnea	Pleomorphic adenoma	Patient reported relief from sleep apnea symptoms, postoperatively	

Sajid et al. (2015) [26]	47/M	>7 years	Right	26 × 20 cm	Large, nodular preauricular mass extending up to submandibular region inferiorly and anteriorly up to 2 cm posterior to the nasolabial fold	MRI showed a heterogeneous, lobulated mass in the superficial lobe of the parotid gland, extending medially up to sternomastoid and carotid sheath. FNAC was indicative of PA	Superficial parotidectomy with excision of redundant skin	22 × 24 × 12 cm (1.8 kg)		Pleomorphic adenoma		

Tarsitano et al. (2015) [27]	83/M	>30 years	Left	35 × 28 cm	Giant, multinodular, pedunculated mass in the preauricular region, extending up to the cervical region	MRI showed a giant, heterogeneous mass arising from the superficial lobe of the parotid gland, with well-demarcated boundaries and preservation of surrounding tissue planes. CT revealed primary blood supply though facial artery and numerous small feeder vessels. Incisional biopsy confirmed the diagnosis of PA	Extracapsular dissection of the tumor mass	33 × 27 × 16 cm (7.3 kg)	The mass became too big and a hindrance for the patient to ambulate	Pleomorphic adenoma		5-year recurrence-free follow-up

Akintububo et al. (2016) [4]	60/M	>10 years	Left	25 × 23 × 17 cm	Giant, lobulated, and pedunculated mass in the preauricular region, extending up to cervical region, with firm consistency and measuring almost the size of the patient's head	CT showed a large soft tissue mass arising from the superficial lobe of the parotid gland and presenting with several amorphous calcifications, but without any bony involvement. FNAC was indicative of PA	Superficial parotidectomy	(5.5 kg)	Patient had delayed surgery due to financial constraints	Pleomorphic adenoma with multiple foci of microcalcifications	Reactionary hemorrhage in the immediate postoperative period, managed through exploratory ligation. Transient neurological deficit of the buccal branch of facial nerve	6-month recurrence-free follow-up

Calvo-Henriquez et al. (2016) [28]	72/F	14 years	Right	20 × 15 cm	Giant preauricular mass, fixed to underlying tissues and associated with facial nerve deficit (House-Brackman Grade III)	CT showed a well-defined parotid mass with mixed solid and cystic areas. The mass extended superiorly up to temporal muscle and cervically up to the hyoid bone. FNAC was indicative of mixed tumor of salivary gland origin	Extracapsular dissection of tumor mass along with a superficial skin island	(1.6 kg)			Facial nerve deficit (House-Brackman Grade III) persisted postoperatively	

Swain (2016) [29]	92/M	>25 years	Right	20 × 15 cm	Large, multinodular preauricular mass with skin ulceration due to repeated trauma for past 6 months	CT showed a well-defined mass involving the superficial lobe of the parotid gland. FNAC was indicative of PA	Superficial parotidectomy with preservation of facial nerve branches		Skin ulceration due to repeated trauma for past 6 months	Pleomorphic adenoma		

Chao et al. (2017) [30]	83/F	>20 years	Right		Patient presented with a slow-growing, preauricular mass 1 year ago which was provisionally identified as PA through FNAC. Patient however delayed surgery due to personal reasons. Rapidly proliferating exophytic, firm, multilobulated growth with skin ulceration and bleeding were seen in the same lesion in the last 1 year. Facial nerve function was intact	CT showed a heterogeneous parotid mass measuring 9 × 8.4 cm with foci of necrosis and calcification. The mass was in close proximity to inferior aspect of external auditory canal and was invading sternomastoid and masseter muscles. Superficially skin erosion was seen. No evidence of lymph node or bony involvement. FNAC was suggestive of carcinoma ex-PA	En bloc resection with total parotidectomy. Facial nerve branches except the buccal branch were preserved. The buccal branch was encased in tumor. Selective neck dissection (levels I-III) along with resection of infratemporal and parapharyngeal spaces. Soft-tissue reconstruction with anterolateral thigh free flap	10.5 cm diameter	Rapid growth with skin ulceration in the last 1 year	True malignant mixed tumor with extensive foci of necrosis and poorly differentiated adenocarcinoma and chondrosarcoma components. No histological evidence of original PA seen	Postoperative radiotherapy 60 Gy	At 3-year follow-up, no loco regional recurrence was observed. Patient however presented with lung and liver nodules on PET scan

Alnofaie et al. (2020) [31]	25/F	10 years	Right	25 × 25 × 15 cm	Irregular, multilobulated, giant mass with a sessile base in the right parotid region extending to the neck. Overlying skin appeared erythematous with prominent vasculature and no discharge. Swelling was fixed to underlying tissues and facial nerve function was unaffected	CT showed a heterogeneous mass lesion arising from the parotid. MRI showed a lobulated heterogeneous mass with multilocular cystic changes and measuring 12 × 10 × 12 cm. No extension to retromandibular or parapharyngeal spaces. FNAC was suggestive of PA	Superficial parotidectomy with preservation of facial nerve branches	18 × 17 × 11.5 cm (1.5 kg)	Patient was mentally challenged and patient's mother requested surgery as the mass had become too large and hindered daily activities	Pleomorphic adenoma		1-year recurrence-free follow-up

Pareek et al. (2020) [32] (case series comprising 15 giant parotid PA)	30-81 years (mean—50.3)/5 males and 10 females	5-20 years	Right—9; left—6	All lesions were greater than 10 cm in diameter (range 10-25 cm)	Majority of the lesions presented as irregular or ovoid parotid masses with well-defined margins and overlying skin was ulcerated in 2 cases. None of the cases had preoperative facial nerve weakness	A combination of USG, CT, MRI, and FNAC to arrive at a provisional diagnosis of PA. One case was preoperatively diagnosed with malignant change based on FNAC	Total parotidectomy—10; total parotidectomy + neck dissection—1; superficial parotidectomy—3; enucleation—1. Facial nerve preserved in all cases	(2.0-3.5 kg; mean—2.7)	Majority of the patients delayed surgery due to poor awareness and underprivileged socioeconomic status	All cases were histologically confirmed as PA, except one case which showed a malignant change	Transient facial nerve deficit in 2 patients which recovered within 6 months. Postoperative radiotherapy only for the malignant case	Minimum 6-month recurrence-free follow-up

M: male/F: female; CT: computed tomography; MRI: magnetic resonance imaging; FNAC: fine-needle aspiration cytology; PA: pleomorphic adenoma; USG: ultrasonography; PET: positron emission tomography.
